# Regular Voluntary Exercise Potentiates Interleukin-1*β* and Interleukin-18 Secretion by Increasing Caspase-1 Expression in Murine Macrophages

**DOI:** 10.1155/2017/9290416

**Published:** 2017-01-04

**Authors:** Ken Shirato, Kazuhiko Imaizumi, Takuya Sakurai, Junetsu Ogasawara, Hideki Ohno, Takako Kizaki

**Affiliations:** ^1^Department of Molecular Predictive Medicine and Sport Science, Kyorin University School of Medicine, 6-20-2 Shinkawa, Mitaka, Tokyo 181-8611, Japan; ^2^Faculty of Human Sciences, Waseda University, 2-579-15 Mikajima, Tokorozawa, Saitama 359-1192, Japan; ^3^Social Medical Corporation, The Yamatokai Foundation, 1-13-12 Nangai, Higashiyamato, Tokyo 207-0014, Japan

## Abstract

Moderate-intensity regular exercise improves proinflammatory responses of lipopolysaccharide- (LPS-) stimulated macrophages. However, intracellular events that mediate the beneficial effects of exercise were unclear. This study aimed to clarify the mechanism by which regular voluntary exercise (VE) improves proinflammatory cytokine production by macrophages challenged with LPS. Peritoneal macrophages from VE mice secreted considerably higher amounts of interleukin- (IL-) 1*β* and IL-18 than did cells from sedentary control (SC) mice in the presence and absence of LPS, although tumor necrosis factor-*α* and IL-10 secretion were comparable between both groups. The mRNA levels of these cytokines increased significantly in response to LPS; similar levels were noted in macrophages from both SC and VE mice. Moreover, LPS evoked similar levels of degradation of inhibitor of *κ*B (I*κ*B) *α* and phosphorylation of I*κ*B kinase *β*, c-Jun N-terminal kinase, and p38 in macrophages from SC and VE mice. These results indicate that the increased IL-1*β* and IL-18 secretion in VE mice are regulated posttranscriptionally. On the other hand, macrophages from VE mice showed higher amounts of caspase-1 protein than did cells from SC mice. These results suggest that regular VE potentiates IL-1*β* and IL-18 secretion in LPS-challenged macrophages by increasing caspase-1 levels.

## 1. Introduction

Macrophages play crucial roles in the first line of host defense against pathogen infection, partially by facilitating inflammation in infectious foci. Macrophages recognize a wide variety of pathogen-associated molecular patterns (PAMPs) such as lipopolysaccharide (LPS), an outer membrane component of gram-negative bacteria, via pattern recognition receptors, including the Toll-like receptor (TLR) family of proteins [1]. TLR4 mediates LPS-induced activation of nuclear factor-*κ*B (NF-*κ*B) and expression of inflammatory cytokines. Activation of NF-*κ*B is followed by phosphorylation of inhibitor of NF-*κ*B (I*κ*B) by the I*κ*B kinase (IKK) complex and its ubiquitination and proteasomal degradation [1]. In addition to NF-*κ*B signaling, mitogen-activated kinase (MAPK) signaling molecules, including c-Jun N-terminal kinase (JNK) and p38, are also activated in response to LPS [2, 3].

Processing of proinflammatory cytokines after translation also influences the degree of inflammation. In particular, the regulatory machinery for interleukin- (IL-) 1*β* and IL-18 maturation and secretion substantially differs from that for most other pro- and anti-inflammatory cytokines, such as tumor necrosis factor- (TNF-) *α* and IL-10 [4]. IL-1*β* and IL-18 are initially synthesized as leaderless precursors that require cleavage into active forms by inflammasome-activated caspase-1 [5, 6]. Danger-associated molecular patterns (DAMPs), for example, ATP, cholesterol crystals, urate crystals, and ceramide, promote formation of the inflammasome, which is a multimeric protein complex consisting of Nod-like receptor family pyrin domain containing 3 (NLRP3), apoptosis-associated speck-like protein containing a caspase recruitment domain (ASC), and procaspase-1 [7–10]. Inflammasome formation leads to cleavage of procaspase-1 to form two active subunits, p20 and p10 [7–10].

Moderate-intensity regular exercise improves the production of proinflammatory cytokines in LPS-stimulated macrophages [11–13]. Our group previously reported that treadmill exercise for 3 weeks causes downregulation of *β*_2_-adrenoceptor expression concomitantly with augmentation of LPS-induced IL-12 production in murine peritoneal macrophages [11]. Gene knock-down and overexpression analyses for *β*_2_-adrenoceptor using murine macrophage RAW264.7 cells have revealed that the receptor negatively regulates intracellular signaling and target gene expression downstream of TLRs [11, 12, 14]. Another group also reported that voluntary wheel running exercise for 8 weeks augments LPS-stimulated secretion of IL-1*β* in murine peritoneal macrophages [13]. However, all these reports lacked evidence on how endurance-type regular exercise modulates intracellular signaling downstream of TLR4 in macrophages immediately after LPS recognition.

Growing evidence indicates that exercise modulates inflammatory cytokine production after the posttranscriptional level. For instance, acute exhaustive exercise was found to suppress LPS-induced expression of TNF-*α* at the protein but not mRNA level in murine liver, lung, and spleen tissues [15]. A recent study showed the importance of genome-wide translational analyses in investigating the effects of exercise on macrophage inflammatory responses [16]. Therefore, to clarify the mechanisms by which moderate-intensity regular exercise improves proinflammatory cytokine production by macrophages in response to LPS, the current study explored the effects of voluntary exercise (VE) for 8 weeks on LPS-induced production of pro- and anti-inflammatory cytokines and activation of intracellular signaling downstream of TLR4, including NF-*κ*B and MAPK signaling, in peritoneal macrophages. Furthermore, we analyzed the levels of inflammasome components to estimate regulatory mechanisms for potentiation of IL-1*β* and IL-18 secretion by regular VE.

## 2. Materials and Methods

### 2.1. Mice and Regular VE

Four-week-old male C57BL/6J mice (*N* = 16; Sankyo Labo Service, Tokyo, Japan) were randomly divided into the sedentary control (SC; *n* = 8) and VE (*n* = 8) groups. The SC mice were individually housed in plastic cages for 8 weeks, whereas the VE mice were individually housed in plastic cages with free access to a running wheel (diameter, 14 cm; width, 6 cm; Melquest, Toyama, Japan) 24 h/day for the same periods as SC mice [17]. The wheels were attached to a permanent magnetic switch that activated a digital counter for wheel revolutions [17]. Total wheel revolutions were recorded daily, with the total distance run per day being determined by multiplying the number of wheel rotations by wheel circumference [17]. Both groups of mice were reared at a temperature of 23°C with a fixed light and dark cycle (light at 7:00–19:00 and dark at 19:00–7:00). Food and water were available ad libitum.

The present study was approved by the Experimental Animal Ethics Committee of Kyorin University (number 187, 2016). It was conducted according to the Guiding Principles for the Care and Use of Animals in the Field of Physiological Sciences approved by the Council of the Physiological Society of Japan and as per the Declaration of Helsinki, 1964. The experiments were performed with the least possible pain or discomfort to the mice.

### 2.2. Isolation and Culture of Peritoneal Macrophages

Four days before isolation of peritoneal cells, 2 mL of 4.05% thioglycollate medium (Becton, Dickinson and Company, Franklin Lakes, NJ, USA) was injected intraperitoneally into the mice [18, 19]. Twenty-four hours after cessation of VE, which was conducted over 8 weeks, the mice were euthanized by CO_2_ inhalation, and the cells were then harvested by sterile lavage of the peritoneal cavity with ice-cold Dulbecco's modified Eagle's medium (DMEM; Sigma-Aldrich, St. Louis, MO, USA). The cells were washed once with ice-cold DMEM and resuspended with DMEM containing 10% heat-inactivated fetal bovine serum (BioWest, Nuaillé, France), 100 units/mL penicillin (Nacalai Tesque, Kyoto, Japan), and 100 *μ*g/mL streptomycin (Nacalai Tesque) to prepare a cell suspension with a density of 1 × 10^6^ cells/mL. Then, they were cultured for 1 h to allow macrophages to adhere to culture dishes or multiwell plates. Immediately after the nonadherent cells were removed, some of the cells were extracted to obtain total cellular RNA or whole cellular proteins; other cells were cultured for 20 min or 6 h in the presence or absence of 100 ng/mL LPS (Sigma-Aldrich).

### 2.3. Real-Time Polymerase Chain Reaction

Total cellular RNA was extracted using the RNAiso Plus reagent (TaKaRa Bio, Shiga, Japan). One microgram of total cellular RNA was converted to single-stranded cDNA using the PrimeScript 1st Strand cDNA Synthesis Kit (TaKaRa Bio). The cDNA (1 *μ*L) was amplified using the FastStart Universal Probe Master (Roche Life Science, Indianapolis, IN, USA) in the 7500 real-time PCR system (ThermoFisher Scientific, Waltham, MA, USA). The polymerase chain reaction (PCR) conditions were as follows: 50°C for 2 min; 95°C for 15 s; and 45 cycles each of 95°C for 15 s and 60°C for 1 min. Information regarding the primers and Universal Probes (Roche Life Science) is provided in Table 1. The mRNA expression levels of the target genes, F4/80, TLR4, IL-1*β*, IL-18, TNF-*α*, IL-10, NLRP3, and caspase-1 were compared with those of 18S rRNA, which was used as an internal control.

### 2.4. Enzyme-Linked Immunosorbent Assay

The cell culture supernatants were collected, centrifuged at 220 ×g for 5 min, and stored at −80°C for later use. The IL-1*β*, IL-18, TNF-*α*, and IL-10 concentrations were measured using the Quantikine Mouse IL-1*β* ELISA Kit (R&D Systems, Minneapolis, MN, USA), Mouse IL-18 ELISA Kit (Medical & Biological Laboratories, Nagoya, Japan), Quantikine Mouse TNF-*α* ELISA Kit (R&D Systems), and Quantikine Mouse IL-10 ELISA Kit (R&D Systems), respectively.

### 2.5. Western Blotting

Whole cellular proteins were extracted using RIPA Buffer (Sigma-Aldrich) supplemented with the Complete Protease Inhibitor Cocktail Tablet (Roche Life Science) and Phosphatase Inhibitor Cocktail (Nacalai Tesque). The concentrations of proteins in the extracts were determined using the BCA Protein Assay Kit (Pierce Biotechnology, Rockford, IL, USA). Protein samples (10 *μ*g) were denatured by incubating them at 95°C for 5 min in sample buffer containing 75 mM Tris-HCl (pH 6.8), 0.6% sodium dodecyl sulfate, 15% glycerol, 7.5%  *β*-mercaptoethanol, and 9 *μ*g/mL bromophenol blue. The denatured proteins were separated by electrophoresis on a sodium dodecyl sulfate-polyacrylamide gel and then transferred onto a polyvinylidene difluoride membrane (GE Healthcare, Buckinghamshire, UK). Each membrane was blocked with 5% bovine serum albumin (Sigma-Aldrich) and incubated with primary antibodies against IL-1*β*, I*κ*B*α*, glyceraldehyde 3-phosphate dehydrogenase (GAPDH), phospho-IKK*β* (Ser177/181), IKK*α*/*β*, phospho-JNK (Thr183/Thr185), JNK, phospho-p38 (Thr180/Tyr182), p38, NLRP3, caspase-8 (Cell Signaling Technology, Danvers, MA, USA), pro-IL-18 (Medical & Biological Laboratories), ASC (Santa Cruz Biotechnology, Dallas, TX, USA), or caspase-1 p20 (AdipoGen, San Diego, CA, USA) at a dilution of 1/1,000. The secondary antibodies horseradish peroxidase-conjugated AffiniPure Mouse Anti-Rabbit IgG or Goat Anti-Mouse IgG (Jackson ImmunoResearch Laboratories, West Grove, PA, USA) were used at a dilution of 1 : 20,000. The membranes were incubated with the Clarity Western ECL Substrate (Bio-Rad Laboratories, Hercules, CA, USA) and exposed to X-ray films. The densities of protein bands were quantified using the ImageJ software (available from the National Institutes of Health, Bethesda, MD, USA).

### 2.6. Immunoprecipitation

Antibodies against NLRP3 (Cell Signaling) or caspase-1 p20 (AdipoGen) were incubated with protein G Sepharose (GE Healthcare) overnight at 4°C. After the redundant antibodies were washed out, the whole cell proteins (400 *μ*g) or cell culture supernatants (1 mL) were incubated with the complex overnight at 4°C. The resultant immune complexes binding to protein G Sepharose were washed thoroughly five times with ice-cold Dulbecco's phosphate-buffered saline supplemented with 0.1% Nonidet P-40. The immunoprecipitated proteins were eluted and denatured by boiling them at 95°C for 5 min in the sample buffer and separated as described above; subsequently, the amounts of intracellular NLRP3 or secreted caspase-1 p20 were analyzed by western blotting.

### 2.7. Macrophage Treatment with Caspase-1 Inhibitor

To elucidate the contribution of caspase-1 activity in the IL-1*β* and IL-18 secretion, peritoneal-exudate macrophages isolated from 10-week-old male C57BL/6J mice (*n* = 3) were stimulated with 100 ng/mL LPS (Sigma-Aldrich) for 6 h in the absence or presence of a caspase-1 inhibitor Ac-YVAD-cmk (Sigma-Aldrich). Ac-YVAD-cmk was dissolved in dimethyl sulfoxide (Wako Pure Chemical Industries, Osaka, Japan) to generate a 100 mM solution, and then this solution was added to the complete medium to achieve a final concentration of 10 *μ*M. The final volume of dimethyl sulfoxide in the medium was equivalent (0.01%) between cells treated with and without Ac-YVAD-cmk.

### 2.8. Statistical Analysis

Experimental data have been presented as mean ± standard error of the mean (SEM). The differences between two groups were assessed using Student's *t*-test. Comparisons among at least three groups were tested by one-way analysis of variance (ANOVA); subsequently, post hoc comparisons were performed to determine significant differences between groups by using the Bonferroni test. Differences were considered statistically significant when *p* values were less than 0.05.

## 3. Results

### 3.1. Regular VE Potentiated IL-1*β* and IL-18 Secretion by Macrophages

**Figure 1:**
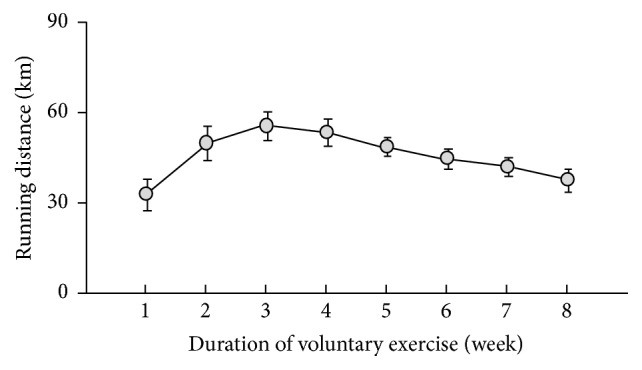
Running distance of the voluntary exercise group mice. Integrated running distance per week during the experimental periods was plotted.

**Figure 2:**
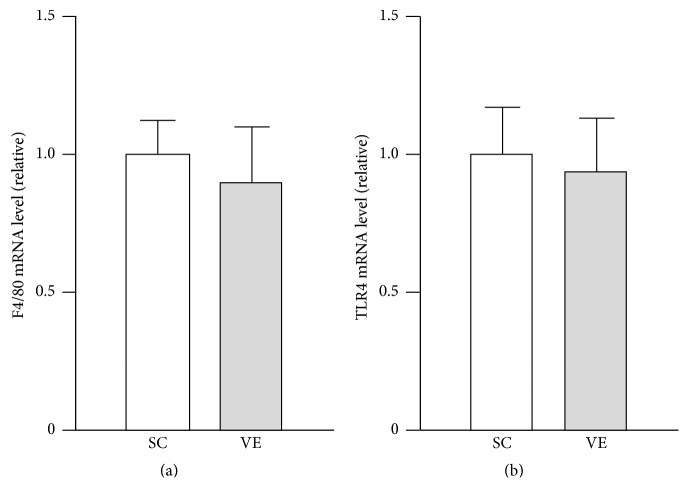
Effects of regular voluntary exercise (VE) on the mRNA levels of F4/80 and TLR4 in macrophages. Immediately after peritoneal-exudate macrophages were isolated from the sedentary control (SC) and VE mice, total cellular RNA were extracted. The mRNA levels of F4/80 (a) and TLR4 (b) were analyzed by real-time polymerase chain reaction (PCR). The mRNA levels are presented as ratios relative to the levels of 18S rRNA. Mean ± standard error of the mean (SEM; *n* = 4) values are provided.

**Figure 3:**
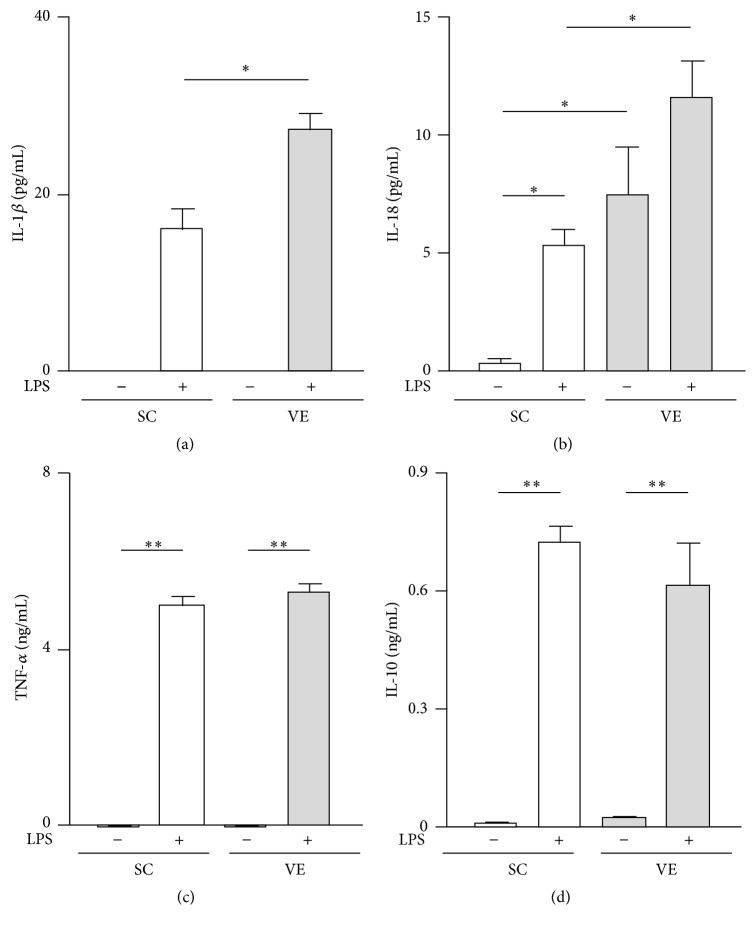
Effects of regular voluntary exercise (VE) on the secretion of pro- and anti-inflammatory cytokines from macrophages stimulated with or without lipopolysaccharide (LPS). Peritoneal-exudate macrophages isolated from the sedentary control (SC) and VE mice were cultured for 6 h in the presence or absence of 100 ng/mL LPS. Concentrations of interleukin- (IL-) 1*β* (a), IL-18 (b), tumor necrosis factor- (TNF-) *α* (c), and IL-10 (d) in the cell culture supernatants were quantified by enzyme-linked immunosorbent assay (ELISA). Mean ± standard error of the mean (SEM; *n* = 4) values are provided. ^*∗*^*p* < 0.05; ^*∗∗*^*p* < 0.01 (by one-way analysis of variance [ANOVA] and the Bonferroni test).

Running distance of the VE mice was improved up to 3 weeks after initiating exercise and then gradually reduced until exercise cessation (Figure 1). Peritoneal injection did not impair activities of the VE mice (Figure 1). We found that the mRNA levels of F4/80 and TLR4 in peritoneal-exudate macrophages did not significantly differ between the SC and VE mice (Figure 2), which suggested that the proportion of macrophage populations recruited into the peritoneal cavity was not affected by regular VE for 8 weeks. We next examined the effects of regular VE on the secretion of pro- and anti-inflammatory cytokines in macrophages. After 6 h culture, the macrophages isolated from the VE mice secreted significantly higher amounts of the proinflammatory cytokines IL-1*β* and IL-18 in response to LPS than did cells isolated from the SC mice (Figures 3(a) and 3(b)). IL-18 secretion from macrophages was significantly higher in the VE mice than in the SC mice even in the absence of LPS (Figure 3(b)), although IL-1*β* secretion was not detected in cells without LPS stimulation (Figure 3(a)). Macrophages from both SC and VE mice secreted similar amounts of TNF-*α* (proinflammatory cytokine) and IL-10 (anti-inflammatory cytokine) in response to LPS (Figures 3(c) and 3(d)). These results suggest that IL-1*β* and IL-18 secretion in macrophages is specifically augmented after regular VE.

### 3.2. Regular VE Did Not Affect Target Gene Expression and Intracellular Signaling Downstream of TLR4 in Macrophages

**Figure 4:**
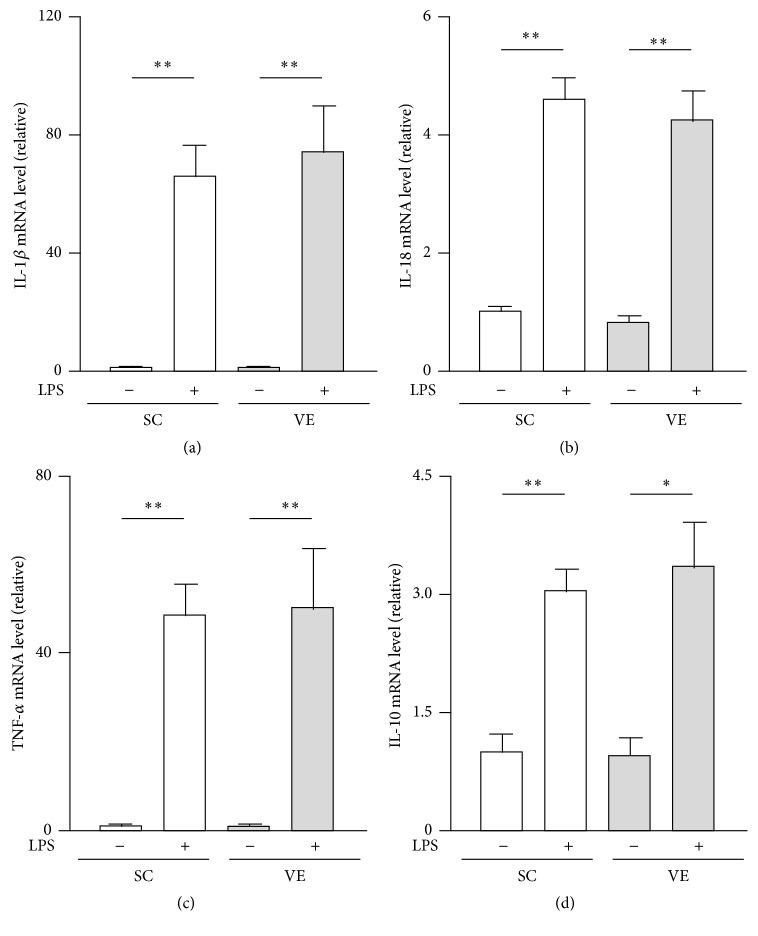
Effects of regular voluntary exercise (VE) on the mRNA levels of pro- and anti-inflammatory cytokines in macrophages stimulated with or without lipopolysaccharide (LPS). Peritoneal-exudate macrophages isolated from the sedentary control (SC) and VE mice were cultured for 6 h in the presence or absence of 100 ng/mL LPS. The mRNA levels of interleukin- (IL-) 1*β* (a), IL-18 (b), tumor necrosis factor- (TNF-) *α* (c), and IL-10 (d) were analyzed by real-time polymerase chain reaction (PCR). The mRNA levels are presented as ratios relative to the levels of 18S rRNA. Mean ± standard error of the mean (SEM; *n* = 4) values are provided. ^*∗*^*p* < 0.05; ^*∗∗*^*p* < 0.01 (by one-way analysis of variance [ANOVA] and the Bonferroni test).

**Figure 5:**
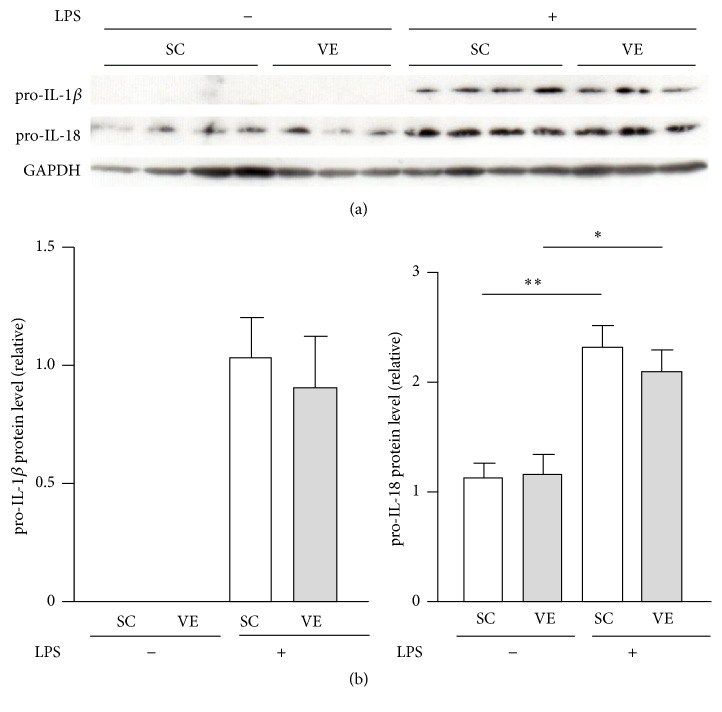
Effects of regular voluntary exercise (VE) on the protein levels of prointerleukin- (IL-) 1*β* and pro-IL-18 in macrophages stimulated with or without lipopolysaccharide (LPS). Peritoneal-exudate macrophages isolated from the sedentary control (SC) and VE mice were cultured for 6 h in the presence or absence of 100 ng/mL LPS. (a) The protein levels of pro-IL-1*β* and pro-IL-18 were analyzed by western blotting. (b) The protein levels are presented as ratios relative to the levels of glyceraldehyde 3-phosphate dehydrogenase (GAPDH). Mean ± standard error of the mean (SEM; *n* = 3-4) values are provided. ^*∗*^*p* < 0.05; ^*∗∗*^*p* < 0.01 (by one-way analysis of variance [ANOVA] and the Bonferroni test).

**Figure 6:**
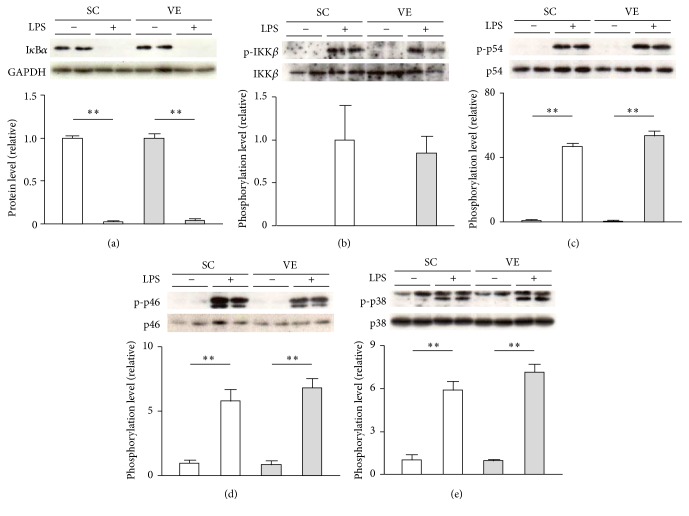
Effects of regular voluntary exercise (VE) on the activation of intracellular signaling downstream of TLR4 in macrophages stimulated with or without lipopolysaccharide (LPS). Peritoneal-exudate macrophages isolated from the sedentary control (SC) and VE mice were cultured for 20 min in the presence or absence of 100 ng/mL LPS. The protein levels of inhibitor of *κ*B (I*κ*B)*α* (a) and phosphorylation levels of I*κ*B kinase (IKK)*β* (b), p54 (c), p46 (d), and p38 (e) were analyzed by western blotting (upper panel). The protein levels are presented as ratios relative to the levels of glyceraldehyde 3-phosphate dehydrogenase (GAPDH) (lower panel), whereas the phosphorylation levels are presented as ratios relative to the respective protein levels (lower panel). Mean ± standard error of the mean (SEM; *n* = 4) values are provided. ^*∗∗*^*p* < 0.01 (by one-way analysis of variance [ANOVA] and the Bonferroni test).

We investigated whether the potentiation of IL-1*β* and IL-18 secretion of macrophages after regular VE is caused by augmented transactivation of their genes and activation of intracellular signaling downstream of TLR4. The mRNA levels of IL-1*β* and IL-18 in macrophages significantly increased after LPS stimulation for 6 h; the levels were not significantly different between the SC and VE mice (Figures 4(a) and 4(b)). A comparable tendency was also observed in the mRNA levels of TNF-*α* and IL-10 (Figures 4(c) and 4(d)). In addition, intracellular protein levels of pro-IL-1*β* and pro-IL-18 were also significantly increased after 6 h culture with LPS, and these levels were not significantly different between the SC and VE mice (Figure 5). Pro-IL-18 was detected in macrophages even in the absence of LPS, although pro-IL-1*β* was not detected in the same condition (Figure 5). Moreover, LPS evoked similar levels of I*κ*B*α* degradation and IKK*β* phosphorylation in macrophages in SC and VE mice after 20 min culture (Figures 6(a) and 6(b)). In addition to these findings for NF-*κ*B signaling molecules, phosphorylation levels of the p54 and p46 subunits of JNK and the p38 MAPK in macrophages also increased to comparable levels in the SC and VE mice in response to LPS (Figures 6(c)–6(e)). These results suggest that regular VE does not affect activation of intracellular signaling downstream of TLR4 after LPS recognition and that the potentiation of LPS-induced IL-1*β* and IL-18 secretion of macrophages after regular VE is regulated at the posttranscriptional level.

### 3.3. Regular VE Increased Intracellular Protein Levels of Pro-Caspase-1 in Macrophages

**Figure 7:**
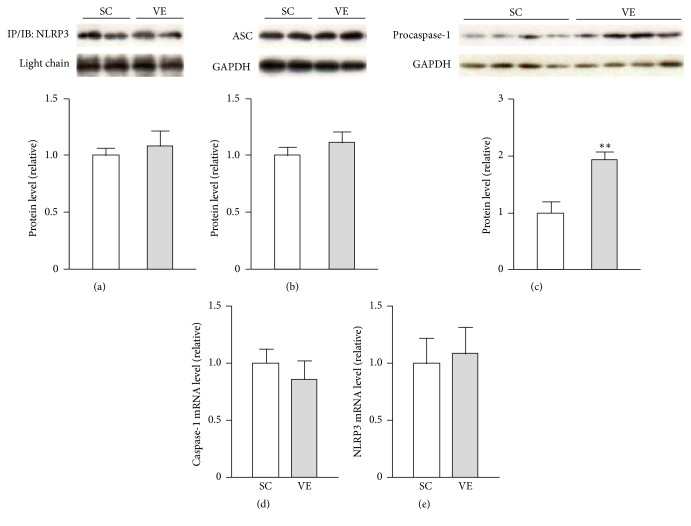
Effects of regular voluntary exercise (VE) on the expression levels of inflammasome components in macrophages. Immediately after peritoneal-exudate macrophages were isolated from the sedentary control (SC) and VE mice, whole cellular proteins and total cellular RNA were extracted. (a) The protein levels of Nod-like receptor family pyrin domain containing 3 (NLRP3) were analyzed by western blotting after immunoprecipitation (IP) (upper panel). The protein levels of apoptosis-associated speck-like protein containing a caspase recruitment domain (ASC) (b) and procaspase-1 (c) were analyzed by western blotting (upper panel). The protein levels are presented as ratios relative to the levels of glyceraldehyde 3-phosphate dehydrogenase (GAPDH) (lower panel). The mRNA levels of caspase-1 (d) and NLRP3 (e) were analyzed by real-time polymerase chain reaction (PCR). The mRNA levels are presented as ratios relative to the levels of 18S rRNA. Mean ± standard error of the mean (SEM; *n* = 4) values are provided. ^*∗∗*^*p* < 0.01 (by Student's *t*-test).

**Figure 8:**
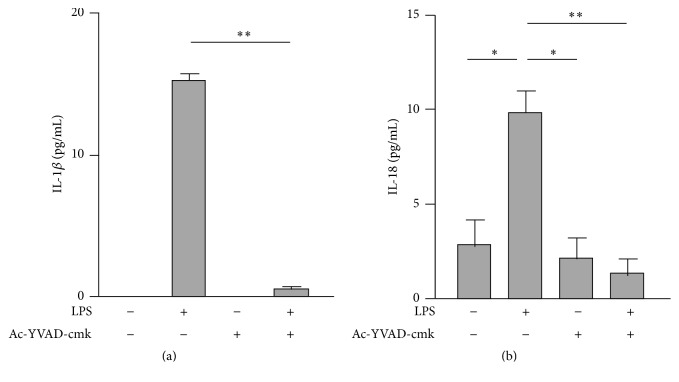
Effects of Ac-YVAD-cmk on the secretion of interleukin- (IL-) 1*β* and IL-18 from macrophages stimulated with or without lipopolysaccharide (LPS). Peritoneal-exudate macrophages were simultaneously treated with 100 ng/mL LPS and 10 *μ*M Ac-YVAD-cmk. Concentrations of interleukin IL-1*β* (a) and IL-18 (b) in the cell culture supernatants were quantified by enzyme-linked immunosorbent assay (ELISA). Mean ± standard error of the mean (SEM; *n* = 3) values are provided. ^*∗*^*p* < 0.05; ^*∗∗*^*p* < 0.01 (by one-way analysis of variance [ANOVA] and the Bonferroni test).

**Figure 9:**
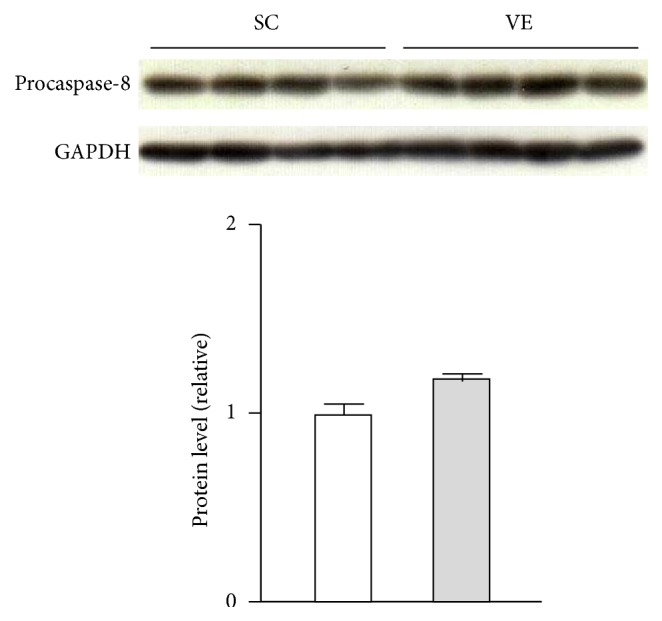
Effects of regular voluntary exercise (VE) on the protein levels of procaspase-8 in macrophages. Immediately after peritoneal-exudate macrophages were isolated from the sedentary control (SC) and VE mice, whole cellular proteins were extracted. The protein levels of procaspase-8 were analyzed by western blotting (upper panel). The protein levels are presented as ratios relative to the levels of glyceraldehyde 3-phosphate dehydrogenase (GAPDH) (lower panel). Mean ± standard error of the mean (SEM; *n* = 4) values are provided.

Maturation and secretion of IL-1*β* and IL-18 after translation are mediated by inflammasome-activated caspase-1 [5, 6]. Therefore, we examined the effects of regular VE on the protein levels of NLRP3 inflammasome components, including NLRP3, ASC, and procaspase-1, in macrophages. Although the NLRP3 and ASC protein levels did not significantly differ between the SC and VE mice (Figures 7(a) and 7(b)), the levels of procaspase-1 were significantly higher in the VE mice than in the SC mice (Figure 7(c)). The mRNA levels of caspase-1 and NLRP3 were comparable between the SC and VE mice (Figures 7(d) and 7(e)). However, secreted forms of procaspase-1 and active p20 subunit could not be detected by western blotting after immunoprecipitation in both the presence and absence of LPS after 6 h culture (see Supplementary Figure 1 in Supplementary Material available online at https://doi.org/10.1155/2017/9290416). To elucidate the contribution of caspase-1 activity in the IL-1*β* and IL-18 secretion, we examined the effects of a caspase-1 inhibitor Ac-YVAD-cmk on the IL-1*β* and IL-18 secretion in peritoneal-exudate macrophages stimulated with or without LPS and found that increased IL-1*β* and IL-18 secretion after 6 h culture with LPS were largely abolished by cotreatment with Ac-YVAD-cmk (Figure 8). Recent findings suggest that caspase-8 also contributes maturation of IL-1*β* [20] and IL-18 [21] in the absence of inflammasome components. To estimate the roles of the inflammasome-independent pathway in the potentiation of IL-1*β* and IL-18 secretion of macrophages after regular VE, we analyzed the protein levels of procaspase-8. However, the levels were not significantly different between the SC and VE mice (Figure 9).

## 4. Discussion

Here, we aimed to determine the mechanism by which regular VE improves proinflammatory cytokine production by macrophages challenged with LPS. The most important findings in this study were that regular VE performed over 8 weeks potentiated IL-1*β* and IL-18 secretion from macrophages (Figures 3(a) and 3(b)) without affecting their mRNA levels (Figures 4(a) and 4(b)) or TLR4-mediated NF-*κ*B and MAPK signaling pathways (Figure 6). Leaderless precursors of IL-1*β* and IL-18 need to be cleaved after translation by inflammasome-activated caspase-1 [5, 6]. It seems, therefore, likely that regular VE influences inflammasome activity in macrophages. Indeed, we found that, among the NLRP3 inflammasome components (NLRP3, ASC, and procaspase-1), the protein levels of procaspase-1 increased in macrophages after regular VE (Figure 7(c)). It is conceivable that VE increased the basal activity of inflammasomes by elevating in the levels of its substrate, procaspase-1, and that the resultant functional caspase-1 potentiated IL-1*β* and IL-18 secretion in macrophages.

However, we could not detect the secretion of its active form p20 subunit from macrophages after regular VE (Supplementary Figure 1). Inflammasome activation is dramatically induced by DAMPs [7–10], which are host-derived molecules that signal the presence of tissue injury [22]. Indeed, p20 secretion could be observed when peritoneal-exudate macrophages were stimulated simultaneously with 100 ng/mL LPS and 20 *μ*M nigericin, an NLRP3 inducer, as with DAMPs (Supplementary Figure 2). Therefore, it is possible that the level of caspase-1 activation in macrophages after regular VE is much less than that noted when the formation of inflammasome complex is dramatically promoted by DAMPs. To support this speculation, we demonstrated that cotreatment of macrophages with a caspase-1 inhibitor Ac-YVAD-cmk abrogated the LPS-induced secretion of IL-1*β* and IL-18 (Figure 8), suggesting that caspase-1 activity mediates IL-1*β* and IL-18 secretion also in DAMPs-free condition. Moreover, although recent studies report that caspase-8 contributes maturation of IL-1*β* [20] and IL-18 [21] in an inflammasome-independent manner, the levels were comparable between the SC and VE mice (Figure 9). It seems, therefore, likely that regular VE specifically increases the protein levels of pro-caspase-1 in peritoneal-exudate macrophages and that the increased procaspase-1 expression is the first candidate to mediate the potentiation of IL-1*β* and IL-18 secretion after regular VE. To further elucidate the contribution of procaspase-1, it is necessary to analyze the mice which genetically lack procaspase-1 in myeloid lineages. Although it is difficult to determine the molecular mechanisms by which regular exercise increases the protein levels of procaspase-1 in macrophages, the increment is regulated at least at the translational level because caspase-1 mRNA levels were not affected by regular VE (Figure 7(d)).

Aberrant inflammation due to dysregulation of inflammasomes is closely associated with immune disorders, including atherosclerosis and bowel diseases [23]. In the present study, although increased secretion of IL-1*β* in macrophages after regular exercise was observed only in the presence of LPS (Figure 3(a)), IL-18 secretion increased under LPS-free as well as LPS-stimulation conditions (Figure 3(b)). The difference in secretion patterns between IL-1*β* and IL-18 can be explained by the results that pro-IL-1*β* expression is LPS-inducible although pro-IL-18 accumulates in peritoneal-exudate macrophages even without LPS stimulation (Figure 5). It is suggested that processing of pro-IL-1*β* is only initiated when its transcription and translation are induced by macrophage recognition of LPS. On the other hand, IL-18 can be secreted without LPS stimulation because detectable amounts of its immature form are already stocked in the cells. A previous study showed that IL-18 is not pyrogenic when injected intraperitoneally in C57BL/6J mice [24]. The researchers also found that IL-18 pretreatment administered 30 min before IL-1*β* attenuated the IL-1*β*-induced febrile response [24]. This characteristic of IL-18 is considered to be mediated by intracellular signaling downstream of IL-18 receptors via p38 instead of NF-*κ*B [25]. Furthermore, it has been reported that IL-18 synergizes with IL-12 for interferon-*γ* production in T lymphocytes and natural killer cells [26, 27], indicating that IL-18 exerts proinflammatory activities predominantly in inflammatory foci. Therefore, it could be considered that, in exercise-trained individuals, macrophages recruited from circulation to inflammatory foci are ready to induce inflammation against pathogens.

Our group and other groups previously reported that regular exercise leads to increased production of proinflammatory cytokines such as IL-12, IL-1*β*, and TNF-*α* in macrophages [11–13]. These proinflammatory phenotypes of macrophages in exercise-trained mice were observed as late-phase events after LPS challenge for 24 h, and intracellular events in the early phase were not investigated [11–13]. In the current study, VE did not potentiate production of TNF-*α* at both the mRNA and protein levels after 6 h culture with LPS and activation of TLR4 signaling after 20 min culture with LPS (Figures 3, 4, and 6). It is possible that the increased IL-1*β* and IL-18 secretion influences the late-phase activation of the proinflammatory responses of macrophages. In particular, increased amounts of IL-1*β* and IL-18 secretion in the early phase after LPS stimulation may activate macrophages by paracrine and/or autocrine actions, leading to potentiation of other cytokines productions in the late phase.

Low-grade chronic systemic inflammation mediated by macrophages that infiltrate into adipose tissues causes insulin resistance and the subsequent pathogenesis of type 2 diabetes mellitus [28]. Growing evidence indicates that exercise training results in switching of the macrophage phenotype from proinflammatory M1 to anti-inflammatory M2 in the adipose and liver tissues in high-fat diet-induced obese mice [29, 30]. However, the current study demonstrated that regular VE did not affect TNF-*α* (Figures 3(c) and 4(c)) and IL-10 (Figures 3(d) and 4(d)) production in macrophages after 6 h culture, in both the presence and absence of LPS. These results indicate that regular exercise does not affect M1 and M2 polarization of macrophages in nonobese mice. This observation is meaningful for host defense, because the induction of inflammation should be critically regulated for an effective host defense to infection [25]. Therefore, regular exercise is beneficial for promoting the proinflammatory responses of macrophages upon pathogen infection in healthy individuals, as well as suppressing dysregulated inflammation by macrophages in obese individuals.

## Supplementary Material

Effects of regular VE on the secretion of pro-caspase-1 and p20 in macrophages stimulated with or without LPS (Supplementary Figure 1).
Effects of nigericin on the secretion of pro-caspase-1 and p20 in macrophages stimulated with or without lipopolysaccharide (LPS) (Supplementary Figure 2).

## Figures and Tables

**Table 1:** Primers and probes used in the real-time PCR.

Protein	Gene	Probe	Forward primer sequence	Reverse primer sequence
F4/80	*Emr1*	#25	5′-TTA TTG TAC GTG CAA CTC AGG ACT-3′	5′-TGG AGC ACT CAT CCA CAT CT-3′
TLR4	*Tlr4*	#2	5′-GGA CTC TGA TCA TGG CAC TG-3′	5′-CTG ATC CAT GCA TTG GTA GGT-3′
IL-1*β*	*Il1b*	#78	5′-TGT AAT GAA AGA CGG CAC ACC-3′	5′-TCT TCT TTG GGT ATT GCT TGG-3′
IL-18	*Il18*	#67	5′-ACA GGC CTG ACA TCT TCT GC-3′	5′-CCT TGA AGT TGA CGC AAG AGT-3′
TNF-*α*	*Tnf*	#49	5′-TCT TCT CAT TCC TGC TTG TGG-3′	5′-GGT CTG GGC CAT AGA ACT GA-3′
IL-10	*Il10*	#21	5′-ACT GCA CCC ACT TCC CAG T-3′	5′-TGT CCA GCT GGT CCT TTG TT-3′
NLRP3	*Nlrp3*	#64	5′-GCT GTG TGA GGC ACT CCA G-3′	5′-GGA GAT GTC GAA GCA GCA TT-3′
Caspase-1	*Casp1*	#27	5′-CGT GAA AGT GAA AGA AAA TCT CAC-3′	5′-GTA CTG TCA GAA GTC TTG TGC TCT G-3′
18S rRNA	*Rn18s*	#55	5′-GGA GAA AAT CTG GCA CCA CAC CTT-3′	5′-CCT TAA TGT CAC GCA CGA TTT CCC-3′
