# Supplementary material for: Regular Voluntary Exercise Potentiates Interleukin-1β and Interleukin-18 Secretion by Increasing Caspase-1 Expression in Murine Macrophages
Source: Mediators Inflamm. 2017 Jan 4;2017:9290416. doi: 10.1155/2017/9290416 (PMC5241476; doi:10.1155/2017/9290416)
Supplement: Supplementary file 1 — Effects of regular VE on the secretion of pro-caspase-1 and p20 in macrophages stimulated with or without LPS (Supplementary Figure 1). Effects of nigericin on the secretion of pro-caspase-1 and p20 in macrophages stimulated with or without lipopolysaccharide (LPS) (Supplementary Figure 2). [file 9290416.f1.docx]

**Regular Voluntary Exercise Potentiates Interleukin-1β and Interleukin-18 Secretion by Increasing Caspase-1 Expression in Murine Macrophages**

**Ken Shirato,^1^ Kazuhiko Imaizumi,^2^ Takuya Sakurai,^1^ Junetsu Ogasawara,^1^ Hideki Ohno,^3^ and Takako Kizaki^1^**

*^1^ Department of Molecular Predictive Medicine and Sport Science, Kyorin University School of Medicine, 6-20-2 Shinkawa, Mitaka, Tokyo 181-8611, Japan*

*^2^ Faculty of Human Sciences, Waseda University, 2-579-15 Mikajima, Tokorozawa, Saitama 359-1192, Japan*

*^3^ Social Medical Corporation, the Yamatokai Foundation, 1-13-12 Nangai, Higashiyamato, Tokyo 207-0014, Japan*

Correspondence should be addressed to Ken Shirato; shirato@ks.kyorin-u.ac.jp

Supplementary Figure 1: Effects of regular voluntary exercise (VE) on the secretion of pro-caspase-1 and p20 in macrophages stimulated with or without lipopolysaccharide (LPS). Peritoneal-exudate macrophages isolated from the sedentary control (SC) and VE mice were cultured for 24 h in the presence or absence of 100 ng/ml LPS. Pro-caspase-1 and p20 were immunoprecipitated from the cell culture supernatants, and then the amounts were analyzed by western blotting. Bands observed clearly at 50 kDa and 25 kDa are heavy and light chains derived from the capsase-1 antibody, respectively. Two bands observed around at 20 kDa are non-specific bands that can be detected by western blotting even without the primary antibody. p20 could not be detected. Although pail bands observed at less than 50 kDa represent pro-caspase-1, it is difficult to be quantified due to their low detectability.

Supplementary Figure 2: Effects of nigericin on the secretion of pro-caspase-1 and p20 in macrophages stimulated with or without lipopolysaccharide (LPS). Peritoneal-exudate macrophages isolated from 12-week-old male C57BL/6J mice were cultured for 6 h in the presence or absence of 100 ng/ml LPS with or without 20 μM nigericin. Pro-caspase-1 and p20 were immunoprecipitated from the cell culture supernatants, and then the amounts were analyzed by western blotting.
